# Interactions between Financial and Environmental Networks in OECD Countries

**DOI:** 10.1371/journal.pone.0136767

**Published:** 2015-09-16

**Authors:** Franco Ruzzenenti, Andreas Joseph, Elisa Ticci, Pietro Vozzella, Giampaolo Gabbi

**Affiliations:** 1 Department of Biotechnology, Chemistry and Pharmacy, University of Siena, Via Aldo Moro 1, IT-53100 Siena, Italy; 2 Advanced Analytics, Bank of England, Threadneedle Street, London EC2R 8AH, United Kingdom; 3 Department of Economics and Statistics, University of Siena, Via S.Francesco 1, IT-53100 Siena, Italy; 4 Department of Business and Law, University of Siena, Via S.Francesco 1, IT-53100 Siena, Italy; Umeå University, SWEDEN

## Abstract

We analysed a multiplex of financial and environmental networks between OECD countries from 2002 to 2010. Foreign direct investments and portfolio investment showing the flows in equity securities, short-term, long-term and total debt, these securities represent the financial layers; emissions of *NO*_*x*_, *PM*10, *SO*_2_, *CO*_2_ *equivalent* and the water footprint associated with international trade represent the environmental layers. We present a new measure of cross-layer correlations between flows in different layers based on reciprocity. For the assessment of results, we implement a null model for this measure based on the exponential random graph theory. We find that short-term financial flows are more correlated with environmental flows than long-term investments. Moreover, the correlations between reverse financial and environmental flows (i.e. the flows of different layers going in opposite directions) are generally stronger than correlations between synergic flows (flows going in the same direction). This suggests a trade-off between financial and environmental layers, where, more financialised countries display higher correlations between outgoing financial flows and incoming environmental flows than from lower financialised countries. Five countries are identified as hubs in this finance-environment multiplex: The United States, France, Germany, Belgium-Luxembourg and United Kingdom.

## 1 Introduction

Network analysis in financial markets has received a growing attention in recent years [1–5]. However, most studies to date have focused on closed systems, such as inter-bank markets [6, 7], ownership networks [8] or networks of directors [9]. Recently network analysis has been applied to cross-border portfolio investment flows showing that network measurements can be used as indicators of structural robustness of financial systems [10]. Research has also been further developed to couple financial networks with real economic networks (cross-border trades, or, the International Trading Network) into an all-inclusive approach [11, 12]. Starting from this latter line of research, the present analysis aims to develop a comparative analysis of the international financial system with the environmental loads carried by goods in the International Trade Network(ITN) within OECD countries.

This study relates to two main strands of the literature: the debate on the interactions between financial and bilateral trade flows [13–15] and that of the nexus between environmental and investment flows, such as foreign direct investment (FDI) [16–18]. Our analysis focuses on correlation and reciprocity structures (the reciprocity is the share of trade that is mutually exchanged among all nodes in a network) of the relations between finance and the environmental content of trades. The environmental content of trades refers to the emissions released, directly and indirectly, locally or globally, by the exporter to produce a certain amount of exported good.

### 1.1 Interaction between finance and real economy: state of art

The existence of reciprocal interaction between financial flows (particularly long term ones, such as FDI) and international trade is well established in the literature [13]. According to the model proposed by Coeurdacier [14], trade openness, namely the exposure of domestic firms to increasing international competition, might foster the acquisition of foreign firms equities as a hedging strategy (i.e, making an investment to reduce the risk of adverse price movements). Consequently, bilateral equity holdings and commercial imports are expected to be positively correlated. Trade relations could also lead to a reduction in borrowing costs which, in turn, stimulates investments. Information asymmetries might provide another explanation: transactions in international trade facilitate information flows between trading partners which, in turn, lowers uncertainties for international financial transactions and vice versa [15]. A growing body of empirical evidence corroborates the hypothesis of the complementarity between trade and FDI [16–18]. The empirical literature has also investigated the correlation between trade and portfolio investments. Aviat and Coeurdacie [15], exploring the geography of trade in goods and asset holdings, for instance, find that the causality between bilateral asset holdings and commercial trade strongly progresses in both ways. Lane and Milesi-Ferretti [19], using data on international portfolio positions, show that there is a strong correlation between bilateral equity holdings and bilateral trade in goods and services. Our study adds to this literature by investigating if this connection between trade and financial flows has environmental implications and whether highly financialised countries have incentives to re-allocate industrial production to less developed countries to reduce their domestic pollution.

Recent contributions on *pollution haven effect* suggested that stringency of environmental regulation affects a country’s competitiveness reducing net exports, increasing net imports and affecting firms’ location choice, and consequently FDI [20–24]. Additionally analyses by Aichele and Felbermayr [25] reveal that the Kyoto Protocol affects trade flows by significantly increasing committed countries’ embodied carbon imports from non-committed countries and the emission intensity of their imports. Less attention is paid to the role for portfolio investments as a channel for higher financialised countries to re-allocate the industrial production into other countries in order to reduce their domestic pollution. In our study, we investigate not only the link between FDI and trade of goods but also the correlation between other financial flows, such as equity and bond securities, which are characterized by a shorter time horizon than FDIs. The research question we want to address is: what role do the long and short term financial flows play with respect to the direction and intensity of international trade and their expected environmental impact? We find that embodied environmental content in imports has, on average, a stronger and more stable correlation and reciprocity with financial outflows, either as FDI and cross-borders portfolio flows, than with inward financial flows. Moreover, this pattern is more accentuated for highly financialised countries. In particular, in the multiplex network of cross-border financial investments and of environmental flows embodied in trade movements, four (France, Germany, USA and UK) out of five hubs are net importers of environmental load. These results cannot definitively detect a causal link but are consistent with the notion that financial markets help the most financialised countries export capitals in exchange of displacement of environmental load.

The rest of the paper is organized as follows. We first present the used data sources and content and we briefly introduce the correlation and reciprocity indexes proposed in the analysis. Then, we discuss the main results and their economic implications. The last section concludes. Supplementary materials and all details of the analyses are reported in S1 File.

## 2 Multiplex finance-environment: analysis and results

### 2.1 Data description and sources

Our network analysis combines various data sources for measures of trade and bilateral financial flows and positions between OECD countries over the 2002–2010 period. More precisely, we consider ten networks where nodes are given by 33 OECD countries and where edges are represented by aggregated/effective cross-border flows of financial investments or goods (see S1 File for a detailed list of nodes and flows). Financial flows comprise bilateral Foreign Direct Investment flows (FDI) and portfolio capital flows. FDI data are reported in current USD millions and come from OECD statistics. Portfolio flows are also measured in current USD millions and data are drawn from the Coordinated Portfolio Investment Survey (CPIS) carried out by IMF [19]. The CPIS records data on the bilateral composition of year-end portfolio holdings (long-term debt, short-term debt and equity portfolio assets) for over seventy reporting/source countries *vis-a-vis* over 200 destination countries, however it does not provide information on portfolio flows. For this reason, all portfolio flows in this study are measured as the difference between consecutive positions an increase (decrease) in debt securities issued by country *j* and held by residents in country *i* is recorded as a capital movement from country *j* to country *i* (from *i* to *j*) [10]. However, it should be noted that taking difference of consecutive investment positions for the CPIS data it is only a proxy for flows, especially for equity, where positions are largely driven by valuation effects.

Earlier studies [19, 26] have already underscored the main limitations of the CPIS in terms of incompleteness, lack of strong data consistence, risk of under-reporting and problematic treatment of intermediated holdings in financial centers. However, the inclusion of portfolio flows, in addition to FDI, allows us to track trends and features of financial investment decisions that are based on different time-horizons and driven by different economic reasons. Moreover, we try to contain some of these problems by focusing the analysis on OECD countries which are more likely to report comprehensive information. We therefore select a set of countries which are expected to ensure the best *trade-off* between data quality and geographical and time coverage. This, however, implies that the interpretation of our results should take into account that large players, such as China, and important resource-rich countries, such as the Gulf States, are excluded from the analysis. As for trade networks, we concentrate on environmental flows which are, directly and indirectly, generated by trade flows. The estimation of the environmental footprint of trade flows draws from the Eora global Multi-Region, Input Output (MRIO) database [27, 28]. Eora elaborates a time series of environmentally extended input-output (IO) tables for 187 countries based on the UN System of National Accounts (SNA), UN COMTRADE, Eurostat, IDE/JETRO, and several national IO tables with matching environmental and social satellite accounts. We refer to this database to obtain the footprint embodied in bilateral import and export flows in terms of five environmental dimensions: emissions of (1) *NO*_*x*_ (in gigagrams, Gg), (2) *PM*10 (Gg), (3) *SO*_2_ (Gg), (4) total *CO*_2_ *equivalent* emissions (Gg) (5) and water footprint (in m3).

### 2.2 Method and analysis

The five financial networks analysed in this study comprise the networks of bilateral foreign direct investment (FDI), portfolio investments in short and long term security debt (SD and LD), in equities (EQ)as well as in total security debts (TD). Environmental and financial networks share the same set of nodes, namely the 33 OECD countries. The entanglement of their relationships gives rise to a *multiplex* of interacting networks [29–31]. Henceforth, every network will be defined as a *layer* of this financial-environmental multiplex.

**Fig 1:**
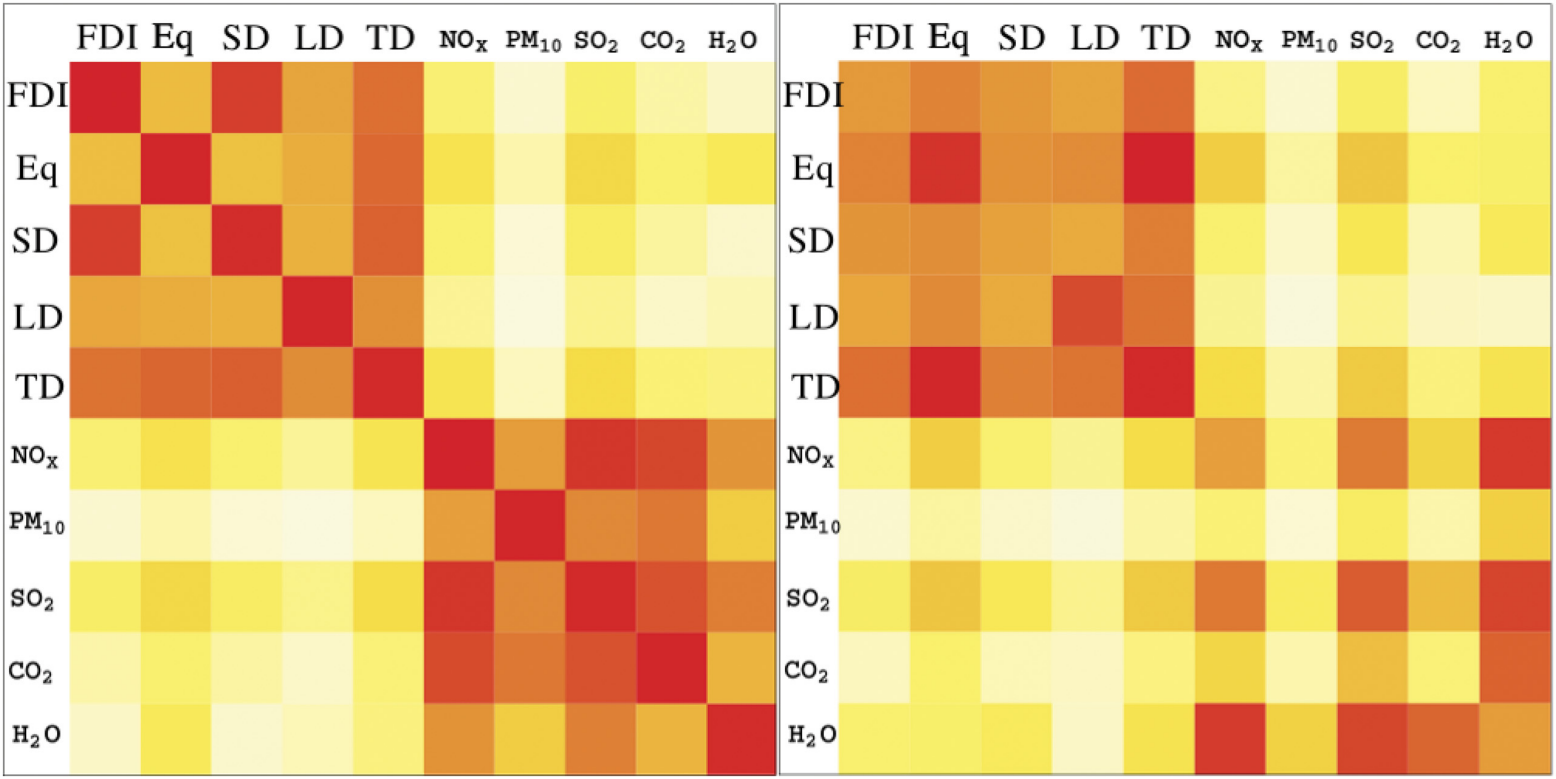
Temperature maps of the Pearson correlation index for synergic (flows in the same direction, left panel) and reverse flows (flows in opposite direction, right panel), 2002–2010. Shade of colours indicate increasing correlation between couple of layers, from light yellow to dark brown. Entries 1–5: FDI, Equity, Short-term Debts, Long-term Debts and Total Debts.

To start with, we investigated the *spatial* correlation among layers by applying a previous method based on the application of the Pearson correlation coefficient to each couple of vertexes in a network [31]. Fig 1 shows the temperature map (colours from white to dark red indicates an increasing, positive correlation) for the five financial layers and the five environmental layers. Correlations are averaged over the period under investigation (2002–2010). Interestingly, but not surprisingly, correlations are all positive: this is due to the fact that the topology in every layer of the multiplex is similar. Hubs of the financial system are also hubs of the ITN (See Tables A and B in S1 File). The first matrix shows correlations between synergic flows, i.e. those going in the same direction, while the second matrix displays correlations of reverse flows correlations, namely those going in opposite direction. Interestingly, the most correlated financial layers to the environmental ones are *equities* and *total debts*. All the environmental layers, correlated more to *equities* (with the exception of reverse flows of water, which correlated more with SD). The second most correlated financial layer to the environment is *TD*. Among the environmental layers, *SO*_2_ is always the most correlated to any financial layer. Although more research would be required on these relationships, our results seem to suggest that financial layers tend to be more correlated trade flows of good produced by manufacturing, metallurgy and, more broadly, by industries relying heavily on raw fossil fuels combustion. The second most correlated is *NO*_*x*_, further suggesting a link to combustion and, foremost, to low-efficiency combustion. The footprint of water is high in the energy intensive and agricultural sectors, and in some manufactures. However, more research is needed. In the remaining part of the article we will investigate to what extent these cross correlations between layers reflect in a specific topology across countries. A second notable result of our analysis is that in most cases (15 out of 25, see Tables C and D in S1 File) and particularly for the most correlated layers (equity, TD, *SO*_2_ and *NO*_*x*_), *reverse* flows display higher correlation to the environment than synergic flows. In other words, among OECD countries financial flows tend to be associated to environmental flows going in the *opposite direction*, more often than the flows going in the *same direction*. Reverse correlations among flows can reflect the presence of reciprocated interactions between financial and environmental flows, that is a tendency of vertex pairs to form mutual connections across different layers. We measure the cross-product, local reciprocity between layer *F* and *E* and country *i* and *j*, where $X_{ij}^{F}$ denotes flows in the layer *F* from country *i* to country *j*, normalised on country’s total exports (∑_*j*_ *X*_*ij*_) [32]:

$$r_{ij}^{FE}=\frac{X_{ij}^{F}X_{ji}^{E}}{\sum_{j}X_{ij}^{F}\sum_{j}X_{ji}^{E}}$$ (1)

**Fig 2:**
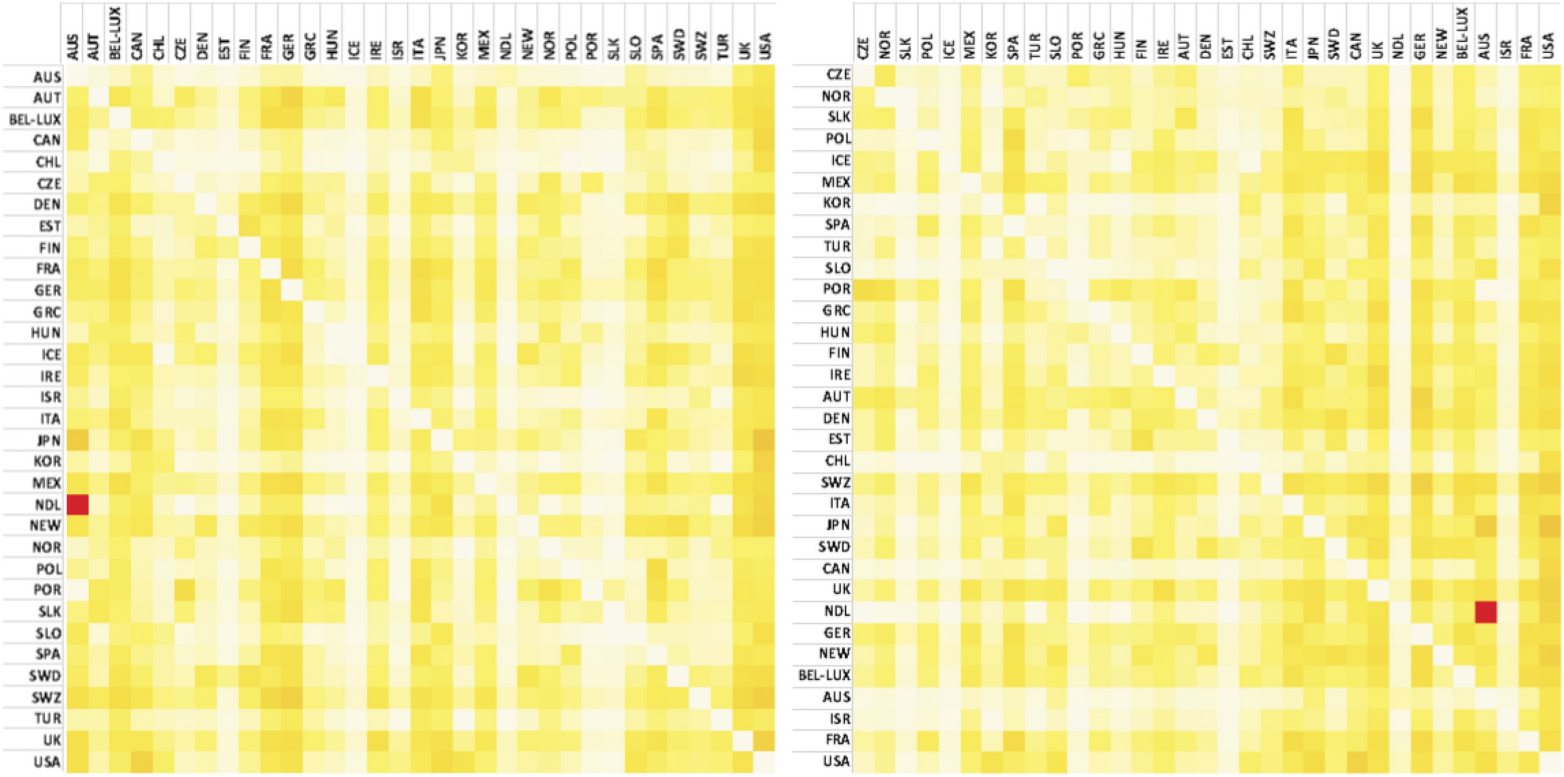
Temperature maps of local reciprocity between financial outflows and environmental inflows (normalised over exports), country panel: countries in alphabetical order, left to right (left panel) and countries ordered for increasing financialisation (right panel), years 2002–2010. Entries are labelled with numbers from 01 to 33 according to Table B in S1 File. From left to right the emerging gradient of yellow indicates that there is a topology in the reciprocity structure that is proportional to the financialisation of countries.

Fig 2 shows the cross-product, local reciprocity (Eq 1) between financial layers (the average $r_{ij}^{FE}$ value of the four financial layers) and environmental layers (the average $r_{ij}^{FE}$ value of the five environmental layers). In the first matrix (Fig 2, left panel), entries (countries) are reported in alphabetical order, while in the second (Fig 2 right panel), countries are ordered by increasing financialisation (measured as value added of the financial sector). Shifting the array-order of countries we can see a gradient of increasing reciprocity emerging from the matrix, when going from less to more financialised countries. This result confirms that there is a specific topology in the financial-environment correlation network linked to the level of countries’ financialisaton. The top-right block of the matrix (Fig 2 right panel) shows on average higher reciprocity than the bottom-left block (see also Table D in S1 File). This asymmetry is evident when looking at the different degrees of yellows in the two regions, indicating a dominant pattern of reciprocity. Focusing on these two blocks which refer to international flows between highly financialised economies and the less financialised one, we can observe that the correlation between financial outflows and environmental imports is stronger for the countries with a high level of financialisation than for the other group. This means that environmental imports correlated more with financial flows from the most to the less financialised countries than with the financial flows in the opposite direction. Moreover, low financialised countries show higher correlation with highly financialised ones than among themselves. Conversely, most financialised countries are tightly connected among themselves. In other words, it seems that highly financialised countries tend to exchange financial flows with environmental flows with countries that are less financialised, predominantly by means of equities and TD.

Given the prevalence of reverse correlations, we can focus our analysis on reciprocal exchanges between the financial and environmental layers. It should be noted that a *multiplex* is a very complex structure [29, 30]. Spurious correlations may rise from overlapping effects driven by two factors: the reciprocity structure within each layer and the topology peculiar of each layer. In the following, we want to disentangle the correlation between each pair of layers from the topology specific to every single layer (with respect to our analysis, reciprocity between layers can be considered as a different form of correlation). By utilising a methodology recently developed in [33, 34], we are able to provide a measure of correlation between layers for reverse flows, on both a global (*ρ* correlations, Eq 14 in S1 File) and local scale (Eq 2) that incorporates a null model and thereby clearing spurious effects from our analysis (for all details see S1 File on local reciprocity and multiplexity):

$$\rho_{ij}^{FE}=\frac{r_{ij}^{FE}-\left\langle r_{ij}^{EE}\right\rangle }{1-\langle r_{ij}^{FE}\rangle }$$ (2)

**Fig 3:**
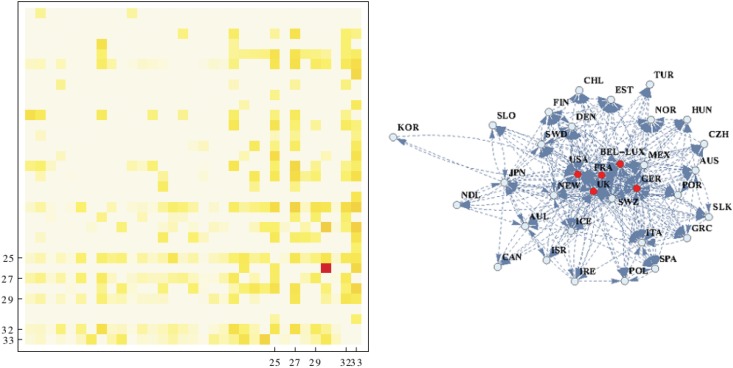
Null-model-enhanced local reciprocity between financial outflows and environmental inflows (local *ρ*); countries ordered by degree of financialisation, left to right (years 2002–2010). Financial flows are outgoing and environmental flows are incoming; exports are normalised over columns (example: entry *w*_*ij*_ indicates the share of financial export of country *j* that is reciprocated by the share of environmental export of country *i*). On the left the temperature map of the reciprocity values that exceed the significance threshold posed by the null model. White dots indicate that there is no significant reciprocity between countries. Yellow dots concentrate in two regions: bottom-right and upper-right quadrants. The first indicate significant reciprocity among highly financialised countries. The second that there is a significant reciprocity from highly financialised countries to less financialised countries. The right panel shows the backbone network of the reciprocity structure: an arrow indicates a link of an outgoing financial flow reciprocated by an incoming environmental flow. The five hub-countries are shown with red dots.

This measure signals when reciprocity exceeds the expected reciprocity trivially produced by the null model. The threshold was calculated by means of *ρ* which scores 0 when the global or local reciprocity between a couple of layers (inter-layer reciprocity) is trivially explained by the null model adopted to explain the topology of every single layer. We expect 0 when a the resulting correlation between layers is only follows as a consequence of the overlapping of two single-layer topologies. The chosen null model is based on an exponential randomization that preserves export, import and reciprocated trade flows for each country pair, which are set as constrains into the model [32, 35]. On the one hand, this null model enables us to test previous results and, on the other hand, to draw out the backbone of significant correlations among countries. According to the cross-product reciprocity and the statistical validation, results of the Pearson index hold: equities and TD correlated more to environmental flows than FDI and reverse correlation tends to prevail over synergic correlation. We can further test the structure of the reciprocal relationship among OECD countries between financial outgoing flows and environmental incoming flows with the null model based on Exponential Random Graph (see S1 File). Fig 3, left panel, shows the reciprocal exchanges between financial and environmental layers exceeding the single-layer reciprocity level set by the null model. The degree of yellow signals positive values of *ρ* whereas the white dots indicates that there is no significant correlation (Eq 2). The top-right block shows, on average, stronger correlations than the bottom-left. This indicates that there are statistically significant correlations between financial outgoing flows reciprocated by incoming environmental flows in the region of the highly financialised and these correlations are stronger than outgoing financial flows from the lowest financialised countries (Table A in S1 File).

**Fig 4:**
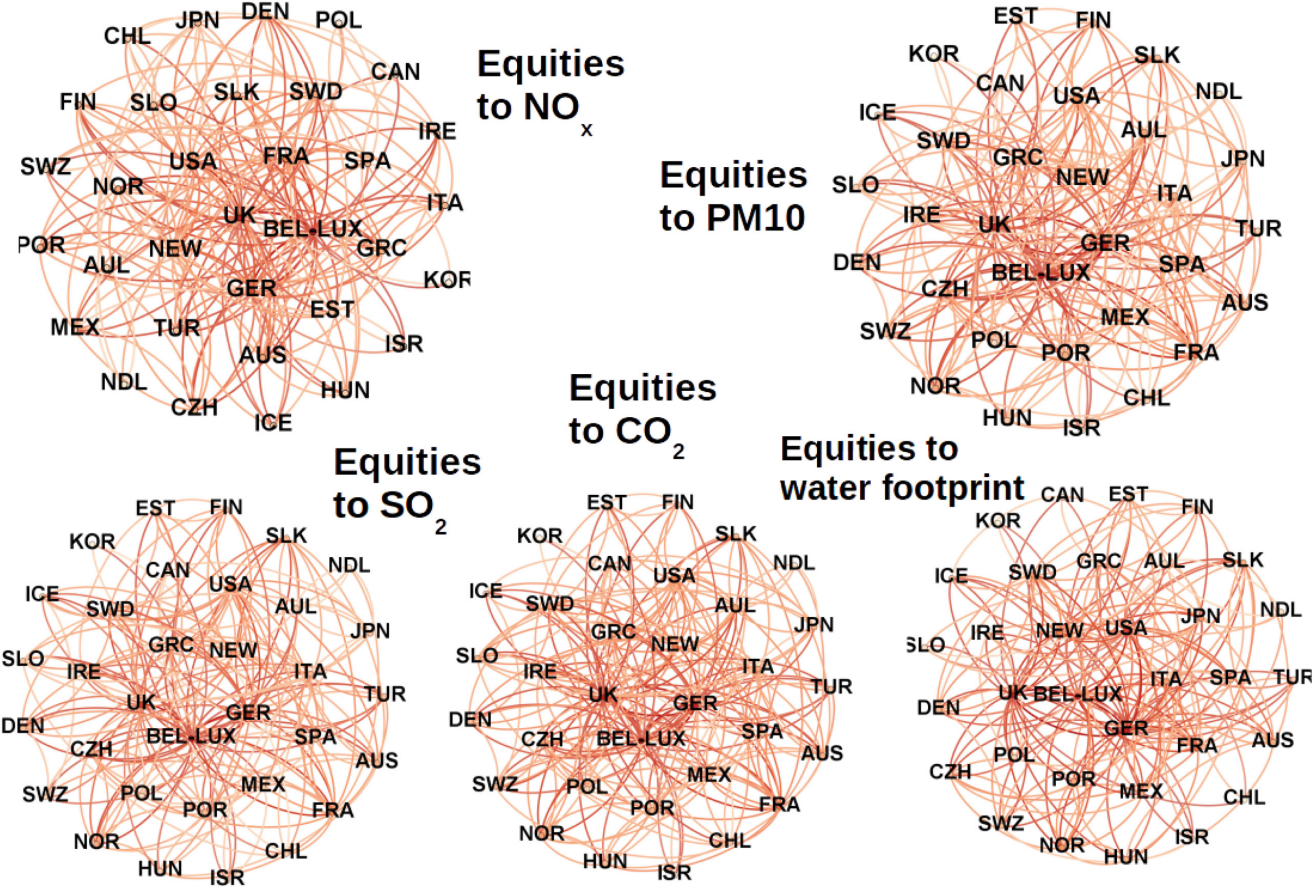
Backbone of the links of null-model-enhanced local reciprocity, between the equity layer and the five environmental layers, for the year 2010: *NO*_*x*_, *PM*10, *SO*_2_, *CO*_2_ *equivalent* and water footprint. Increasing dark red indicate an increasing out-degree of the node. The hubs are placed in the core of the cloud. The reciprocity analysis confirms that equity is mostly reciprocated with *NO*_*x*_ and *SO*_2_, suggesting a link with the industrial sector.

The yellow dots in Fig 3, left panel, can be converted into directed links in order to depict the topology of reciprocal relations among OECD countries. Links map the network backbone of positive correlations exceeding the level of significance posed by the null model. The arrows in the Fig 3 (right panel) stand for a statistically significant outgoing financial flow reciprocated by an incoming environmental flow. For almost all layer pairs and for the financial-environmental layers in aggregate, the backbone of the correlation network highlights the central role of five distinctive nodes: USA, France, Germany, Bel-Lux and UK. Fig 4 shows the relationship between equity and the five environmental layers in these countries. These five latter countries display a number of links much higher than the other OECD countries (See S1 Fig for a detailed counting of links). This observation mirrors the central role that these countries take in both financial markets and international trade. Moreover, contrary to most of the developed and financialised countries, these economies are net importers both of environmental flows (with the exception of Bel-Lux, see S2 Fig) and financial flows (see S3 Fig). The only exception is Germany which is a large exporter of capitals and, interestingly, is a net exporter when trade flows are measured in monetary terms, but it is a net importer if trade flows are evaluated for their environmental content or in mass (for details see Error Analysis in S1 File). This apparent inconsistency can be explained by the fact that industrialized countries transform row materials into composite goods. This explains their negative mass imbalance. Goods that are positioned higher in the value chain have arguably a lesser content of mass and an higher environmental footprint by unit of value. Of course, this is just a supposition based on the evidence that these five reach countries are, in general, net importer of mass and environmental load embodied in goods (see S2 and S4 Figs), but more research is needed.

## 3 Conclusions

In this study, we have focused on the linkage between financial and environmental movements which are generated by cross-border investment activities and the trade of goods, respectively. In contrast to previous studies, we have compared the correlation between environmental and financial flows, both as FDI and as portfolio investment flows. We have found that the environmental footprint embodied in international trade flows, especially measured in terms of SO2 and NOX emissions, correlated more with portfolio investment flows than with FDI. A second finding is that reverse flows, particularly equity investments, show higher correlation with environmental flows than synergic flows, indicating that, among OECD countries, financial flows tend to be associated with environmental flows going in the opposite direction. We have elaborated reciprocity indexes to measure this tendency of country pairs to activate mutual exchanges between environmental and financial networks. Our analysis suggests that the dominant pattern in reciprocated connections between finance and environment is directed from more to less financialised countries. These results open interesting areas of further research as it is coherent with the hypothesis that international financial markets enable countries, especially if highly financialised, to decouple environmental pressure from their economic growth not only through cross-border long-term and stable investments but also through international investments which are more speculative or oriented to short-term returns. The conclusion from this is that agents in net-importer and highly financialised countries tend to take speculative exposures in stocks traded in less industrialized countries. However, not all financialised countries exhibit a significant correlation between financial and environmental layers (see Table C in S1 File for the complete ranking of OECD countries). The back-bone within this highly complex structure is formed by five countries, which are hubs for international trade and investment, namely: the USA, France, Germany, Bel-Lux and the UK. These five countries all but Germany are both net importers of finance and net importers of mass (Calculations on mass balance of every single country were performed on the CEPII BACI data set, see S1 File for further information). Do these results, concerning the topology of correlations, hold even if we would enlarge the scope of the analysis, including emerging economies and developing countries? What is the causal relationship beyond these correlations? Do financial investments draw environmental load or financial flows follow the channels of trade? Although we cannot derive conclusions on causality, we have begun first steps in contributing to this area of research in three main ways: 1) by applying network analysis instruments to study the links between financial flows and trade-related environmental movements (beyond bilateral view); 2) by including portfolio investment flows (beyond FDI); 3) by evaluating trade flows according to their environmental content rather than in monetary or mass terms (beyond disciplinary separatism). What we get is a picture of the world, albeit reduced to only OECD countries, of immense complexity, in which the interacting systems, even those seemingly distinct, can not be seen separately. To say it succinctly, with the stylish and sarcastic words of Oscar Wilde [36]:

London is too full of fogs and serious people. Whether the fogs produce the serious people or whether the serious people produce the fogs, I don’t know.

For more information, see Supplementary Information.

## Supporting Information

S1 File Interactions between financial and environmental networks in OECD countries: Supporting Information File. This file includes Tables A-E. Table A, list of layers of the multiplex. Table B, ranking of OECD countries for FIRE/TV. Table C, average Pearson correlation index of the five financial flows with the five environmental flows. Table D, average reciprocity and multiplexity of five the financial flows with the five environmental flows. Table E, average Pearson correlation of the financial flows with the environment ones, years 2002–2010, without 2008.
(PDF)

S1 Fig Time evolution of number of the number of incoming (left) and (right) outgoing links for synergic flows (*μ* correlations) and the second row for reverse flows (*ρ* correlations). The *y* axis shows the number of links for a given country in the backbone of significant correlations (Fig 3 in the text) between the financial layers, on one side, and environmental layers on the other side. A link is placed between two countries when the *μ* or *ρ* values exceed the threshold value posed by the NM). The colors and the numbers on the curves correspond to the 33 OECD countries.
(EPS)

S2 Fig Time evolution of the imbalance of 5 financial layers (layers 1–5), panel a) and the 5 environmental layers (layers 6–10), panel b), for the five hubs (from top to the bottom): UK, Germany, Bel-Lux, France, USA. (EPS)

S3 Fig Net financial account (BoP, current US dollars) (average 2005–2010). Colours from light yellow to bright red indicate increasing financialisation. Authors’ elaborations based on World Development Indicators accessed in May 2015.
(EPS)

S4 Fig Mass imbalance, (Exp-Imp, tons, layers 1–4) for the 33 OECD countries (average 2002–2010). Colours from light yellow to bright red indicate increasing financialisation. In the region above the line exports in mass exceeds imports. Values are reported on log-10-scale. Source data: CEPII BACI data set.
(EPS)

S5 Fig RWCM null model, predicted and observed topology: plot of expected (green) vs observed (red) links’ weight over the nodes strength (export). (EPS)
