# Supplementary material for: Interactions between Financial and Environmental Networks in OECD Countries
Source: PLoS One. 2015 Sep 16;10(9):e0136767. doi: 10.1371/journal.pone.0136767 (PMC4573761; doi:10.1371/journal.pone.0136767)
Supplement: S1 File — This file includes Tables A-E. Table A, list of layers of the multiplex. Table B, ranking of OECD countries for FIRE/TV. Table C, average Pearson correlation index of the five financial flows with the five environmental flows. Table D, average reciprocity and multiplexity of five the financial flows with the five environmental flows. Table E, average Pearson correlation of the financial flows with the environment ones, years 2002–2010, without 2008. (PDF) [file pone.0136767.s001.pdf]

Interactions between financial and environmental networks in OECD countries: Supporting Information

Franco Ruzzenenti [1,3,⋆], Andreas Joseph [2], Elisa Ticci [1], Pietro Vozzella [4] and Giampaolo Gabbi [4]*

[1] *Department of Economics and Statistics, University of Siena, Via S.Francesco 1, IT-53100 Siena, Italy*
[2] *Advanced Analytics, Bank of England, Threadneedle Street, London EC2R 8AH, UK*
[3] *Department of Biotechnology, Chemistry and Pharmacy, University of Siena, Via Aldo Moro 1, IT-53100 Siena, Italy*
[4] *Department of Business and Law, University of Siena, Via S.Francesco 1, IT-53100 Siena, Italy*
⋆ Corresponding author: ruzzenenti@gmail.com

Abstract

We analysed a multiplex of financial and environmental networks between OECD countries from 2002 to 2010. Foreign direct investments and portfolio investment showing the flows in equity securities, short-term, long-term and total debt, these securities represent the financial layers; emissions of NO_x, $PM10$, SO_2, CO_2 *equivalent* and the water footprint associated with international trade represent the environmental layers. We present a new measure of cross-layer correlations between flows in different layers based on reciprocity. For the assessment of results, we implement a null model for this measure based on the exponential random graph theory. We find that short-term financial flows are more correlated with environmental flows than long-term investments. Moreover, the correlations between reverse financial and environmental flows (i.e. the flows of different layers going in opposite directions) are generally stronger than correlations between synergic flows (flows going in the same direction). This suggests a trade-off between financial and environmental layers, where, more financialised countries display higher correlations between outgoing financial flows and incoming environmental flows than from lower financialised countries. Five countries are identified as hubs in this finance-environment multiplex: The United States, France, Germany, Belgium-Luxembourg and United Kingdom.
Keywords: Finance, Environment, International Trade, Network Theory, Multiplex

1 Multiplex description

What is a *multiplex*? A multiplex is a bundle of networks (layers) that share the *same nodes* but potentially with different flows (edges) within each layer. Each network, or *layer*, can feature relationships of the *same nature* and measured in the same unit or can

*This paper does not represent the views of the Bank of England. The views expressed are those of the authors alone and do not necessarily reflect those of the Bank of England.

Table A. List of multiplex' layers

Financial networks		
FDI	Foreign Direct Investment	1
Equity		2
SD	Short Term debts	3
LD	Long Term debts	4
TD	Total debts	5
Environmenatl networks		
NOx	Nitrogen oxide	6
PM10	Particulate Matter	7
SO2	Sulfur dioxide	8
CO2	Carbon Dioxide	9
Water		10

display a broad variety of connections and, thus, links will be measured in different units [1, 2]. Metrics of our analysis will be of two types: monetary units (USD) for the financial layers and mass units for the environmental layers (Table A). The nodes of the multiplex are 33 OECD countries[1] and will be ordered according to the increasing financialisation of their economy, expressed by value added accounted by financial intermediation, real estate, renting and business activities as a percentage of total value added in 2000, namely prior to the period under study (Table B). We have analysed the interactions of the resulting financial-environmental multiplex over a time span of 9 years, from 2002 to 2010. These multiplex' interactions over 9 years for 10 layers and 33 nodes can be expressed as a tensor σ_{tkij} with dimensions $(9x10x33x33)$ and the entry of the tensor $w(t)^k_{ij}$ denotes the flow of the layer k at time t from country i to country j. The total dataset for this analysis will thus has thus 95040 data points.

2 A measure of correlation: the Pearson correlation index

A first method to investigate layers' correlations in a multiplex framework was proposed by Garlaschelli et al. and applied to the commodity-specific trades [3]. They extended the Pearson correlation index to the multiplex by averaging over space instead of time. The Pearson correlation index between the layers A and B (at time t) will thus be:

$$\rho^{AB}_{Syn} \equiv \frac{\sum_{i\neq j}(w^A_{ij} - \mu^A)(w^B_{ij} - \mu^B)}{\sqrt[2]{\sum_{i\neq j}(w^A_{ij} - \mu^A)^2 (w^B_{ij} - \mu^B)^2}} = \frac{cov_{AB}}{\sigma_A \sigma_B}. \quad (1)$$

It is noteworthy that the above definition of correlation can be applied both to links going in the same direction (equation 1) and to links going in opposite directions (equation

[1]Belgium and Luxembourg are considered together as in the CEPII BACI database concerning trade in mass units these two countries are aggregated. We used CEPII BACI data to analyse mass imbalance of countries and compare this with the environmental flows of their concern.

Table B. List of multiplex' nodes

Country	ranking	FIRE/TV [2]
Czech Republic	1	16.2
Norway	2	16.9
Slovak Republic	3	17.1
Poland	4	18.1
Iceland	5	18.9
Mexico	6	19.0
Korea	7	19.3
Spain	8	19.5
Turkey	9	19.5
Slovenia	10	20.2
Portugal	11	20.3
Greece	12	20.6
Hungary	13	20.9
Finland	14	21.0
Ireland	15	21.3
Austria	16	21.5
Denmark	17	22.3
Estonia	18	22.4
Chile	19	23.1
Switzerland	20	24.0
Italy	21	24.7
Japan	22	24.9
Sweden	23	24.9
Canada	24	25.0
United Kingdom	25	27.0
Netherlands	26	27.3
Germany	27	27.5
New Zealand	28	27.8
Bel-Lux	29	29.1
Australia	30	29.1
Israel	31	30.5
France	32	30.7
United States	33	31.7

2). We call the former *synergic* (*syn*) correlations and the latter *reverse* (*rev*) correlation, see Fig. 1 in the text.

$$\rho_{Rev}^{AB} \equiv \frac{\sum_{i\neq j}(w_{ij}^{A} - \mu^{A})(w_{ji}^{B} - \mu^{B})}{\sqrt[2]{\sum_{i\neq j}(w_{ij}^{A} - \mu^{A})^{2}(w_{ji}^{B} - \mu^{B})^{2}}} \tag{2}$$

Both kinds of correlations, *syn* and *rev*, show that the multiplex is divided in two separate blocks: the financial block (entries 1 to 5), with a weak inner correlation and the environmental block, with a stronger inner correlation. It should be noted that the diagonal elements of the *syn* correlation matrix are 1 by definition, whereas the *rev* correlations on the diagonal score 1 only when the layer's original matrix is symmetrical. Therefore, elements on the diagonal of the *rev* correlation matrix are measures of the symmetry of the single layer matrix. Indeed, this is a hint of the entanglement of reciprocity and correlation in multiplex networks.

Equation 2 can be applied to the binary structure of layers, where the weighted entries w_{ij}^{k} of layer k are replaced by the binary entries a_{ij}^{k} that score 1 when a link exists between node i and j and 0 otherwise. For the binary representation of networks, the Pearson correlation matrix is thus:

$$\rho_{b}^{AB} \equiv \frac{\sum_{i\neq j}(a_{ij}^{A} - \mu^{A})(a_{ij}^{B} - \mu^{B})}{\sqrt[2]{\sum_{i\neq j}(a_{ij}^{A} - \mu^{A})^{2}(a_{ij}^{B} - \mu^{B})^{2}}} \tag{3}$$

The *connectance* (the ratio of existing links over the number of possible links $N(N-1)$, given a network with N nodes, equation 5) of the environmental networks is 1 for every layer, whereas in the financial networks it oscillates around 0.5. It is noteworthy that, in a dense network, mutual links are more likely to occur than in a sparse network and in a fully connected network the reciprocity is *trivially* maximal.

For binary and directed networks, the reciprocity is defined as the fraction of links having a "partner" pointing in the opposite direction:

$$r^{b} \equiv \frac{L^{\leftrightarrow}}{L} \tag{4}$$

where $L = \sum_{i\neq j} a_{ij}$ and $L^{\leftrightarrow} = \sum_{i\neq j} a_{ij}a_{ji}$. The above quantity, r^{b}, is not independent of the link density (or connectance) $c \equiv \frac{L}{N(N-1)} = \frac{\sum_{i\neq j} a_{ij}}{N(N-1)} \equiv \bar{a}$. On the contrary, it can be shown that c is the expected value of r^{b} under the Directed Random Graph Model [8, 7]. In the DRG, a directed link is placed with probability p between any two vertices, i.e. $\langle a_{ij}\rangle_{DRG} = p$, $\forall\, i, j$ (with $i \neq j$). This implies

$$\langle r^{b}\rangle_{DRG} \equiv \frac{\langle L^{\leftrightarrow}\rangle}{\langle L\rangle} = \frac{N(N-1)p^{2}}{N(N-1)p} = p \equiv \frac{L}{N(N-1)} = c \tag{5}$$

showing that the expected value of r^{b} coincides with the fundamental parameter of this null model, and hence depends on L and N. In order to assess whether there is an actual tendency in the network to establish mutual links, one should compare the measured r^{b} with its expected value $\langle r^{b}\rangle_{DRG}$. This means that r^{b} cannot be used to consistently rank networks with different values of L and N, because of their different reference values.

3 Reciprocity as a correlation coefficient

A measure of reciprocity based on the Pearson correlation index was proposed in [7]. Reciprocity is therefore the Pearson correlation coefficient between the transpose elements of the adjacency matrix [9]:

$$\rho^b \equiv \frac{\sum_{i\neq j}(a_{ij}-c)(a_{ji}-c)}{\sum_{i\neq j}(a_{ij}-c)^2} = \frac{r^b - c}{1-c} = \frac{r^b - \langle r^b \rangle_{DRG}}{1-\langle r^b \rangle_{DRG}}. \tag{6}$$

A symmetrical adjacency matrix represents a network with the highest values of r^b and ρ (both equal to 1, thus regarding a fully connected network), whereas a fully asymmetrical one, with *zero* values mirroring *unit* values on opposite sides of the main diagonal (like a triangular matrix), displays the lowest value, being $r^b = 0$ and $\rho = -c/(1-c)$) [7]. This meaningful definition of reciprocity automatically discounts density effects, i.e. the expectation value of r^b. As a result, consistent rankings and temporal analyses become possible in terms of ρ. However, this definition of reciprocity, mutated from Pearson, only works for single binary networks. What would be a definition of reciprocity for a weighted multiplex? First, we will extend the definition of binary reciprocity to weighted networks.

3.1 From binary to weighted

If we follow the binary recipe from left to right, we define the weighted reciprocity as the Pearson correlation coefficient (where, as usual, $\bar{w} = \frac{\sum_{i\neq j} w_{ij}}{N(N-1)} = \frac{W_{tot}}{N(N-1)}$):

$$\rho \equiv \frac{\sum_{i\neq j}(w_{ij}-\bar{w})(w_{ji}-\bar{w})}{\sum_{i\neq j}(w_{ij}-\bar{w})^2} = \frac{r - c^w}{1-c^w} \tag{7}$$

where, in order to produce a result formally equivalent to eq.(6), we have defined the weighted analogues of r and c as follows:

$$r \equiv \frac{\sum_{i\neq j} w_{ij}w_{ji}}{\sum_{i\neq j} w_{ij}^2},\ c^w \equiv \frac{\bar{w}^2}{\sum_{i\neq j} w_{ij}^2/N(N-1)} \tag{8}$$

Note that the equivalence $\bar{a} = c$, valid for the binary case, no longer holds: $\bar{w} \neq c^w$. The previous expressions generalize the binary ones and reduce them when substituting a_{ij} for w_{ij}. In [4], we propose a different measure:

$$r \equiv \frac{W^{\leftrightarrow}}{W} = \frac{\sum_{i\neq j}\min[w_{ij},\ w_{ji}]}{\sum_{i\neq j} w_{ij}}. \tag{9}$$

Indeed, the few attempts that have been made so far in order to characterize the reciprocity of weighted networks [11, 12, 13, 14] are all based on measures of correlation or symmetry between mutual weights.

4 A correlation measure for a multiplex: cross product reciprocity and multiplexity

A multiplex can be featured by homogeneous layers -layers whose nature of trades and thereby unit of account, is the same throughout, or heterogeneous -multiplex composed by layers of different nature and units. The multiplex of the present analysis belongs to the latter case. However, does the definition of reciprocity in equation (9) apply to both cases? Although the definition of reciprocity based on *minimum* (equation 9) is a more refined measure, as provided in [4], it has one major flaw when applied to a multiplex since it is sensible to the scale of layers:

$$r^{AB} = \frac{2\sum_{i\neq j}\min[w_{ij}^{A},\, w_{ji}^{B}]}{\sum_{i\neq j} w_{ij}^{A} + \sum_{i\neq j} w_{ij}^{B}}. \tag{10}$$

This problem becomes stringent when dealing with layers of very different nature and, thus, units. Setting the informative level of reciprocity, based on the minimum amount exchanged, would draw to the arbitrary decision of the scale of the unit. Therefore, we will explore a measure for the multiplex (cross) reciprocity based on equation 8:

$$r^{AB} = \frac{\sum_{i\neq j} w_{ij}^{A} w_{ji}^{B}}{\sum_{i\neq j} w_{ij}^{A} \sum_{i\neq j} w_{ij}^{B}} = \frac{\sum_{i\neq j} w_{ij}^{A} w_{ji}^{B}}{W_{tot}^{A} W_{tot}^{B}} \tag{11}$$

Although equation (11) is less precise to assess the reciprocity of every single layer compared to 9, it is a suitable measure of correlation across layers. We can thereby measure correlation between flows in opposite direction (*reverse* flows) and flows in the same direction (*synergic* flows), for every couple of layers A and B. In the former case, r is a measure of the reciprocity for a multiplex composed by a couple of layers. In the latter case, r is no longer a measure of reciprocity because the layers' flows run in the same direction: we will name this measure *multiplexity*:

$$m_{Syn}^{AB} = \frac{\sum_{i\neq j} w_{ij}^{A} w_{ij}^{B}}{\sum_{i\neq j} w_{ij}^{A} \sum_{i\neq j} w_{ij}^{B}} \tag{12}$$

It is noteworthy that equation (11) is scale free and therefore suitable for our multiplex, that displays different units and scale for each layer. It should be noted that the diagonal elements of the reciprocity matrix, contrary to those of the correlation matrix based on (1), are not equal to 1 by definition. This is due to the fact that equation 11 measures the reciprocity of the multiplex and, thus, scores 1 on the diagonal only when the layer is symmetrical (fully reciprocated).

Furthermore, reciprocity according to equation 11 is easily handled to compute null models, as the expected values of products are the products of expected values, for independent variables:

$$\langle W_{\bigcap}^{AB} \rangle = \sum_{i\neq j} \langle w_{ij}^{A} \rangle \langle w_{ji}^{B} \rangle \tag{13}$$

Hence, a definition of correlation based on cross-products reciprocity and integrated by corresponding null model (as previously shown for the binary case) is:

$$\rho^{AB} = \frac{r^{AB} - \langle r^{AB} \rangle}{1 - \langle r^{AB} \rangle} \tag{14}$$

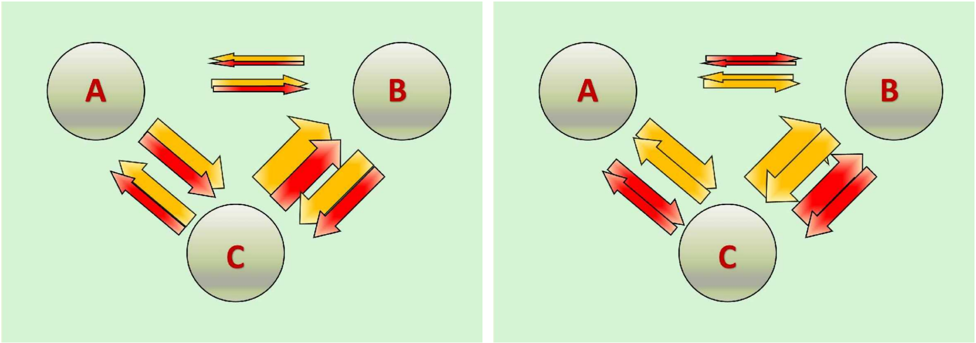

Figure A. Disentangling correlation and reciprocity across layers

Therefore, the μ multiplexity between two layers is:

$$\mu^{AB} = \frac{m^{AB} - \langle m^{AB} \rangle}{1 - \langle m^{AB} \rangle} \tag{15}$$

where

$$\langle r^{AB} \rangle = \frac{\sum_{i \neq j} \langle w_{ij}^A \rangle \langle w_{ji}^B \rangle}{\sum_{i \neq j} \langle w_{ij}^A \rangle \sum_{i \neq j} \langle w_{ji}^B \rangle} \tag{16}$$

and

$$\langle m^{AB} \rangle = \frac{\sum_{i \neq j} \langle w_{ij}^A \rangle \langle w_{ij}^B \rangle}{\sum_{i \neq j} \langle w_{ij}^A \rangle \sum_{i \neq j} \langle w_{ij}^B \rangle} \tag{17}$$

5 Null models

A null model is meant to test the statistics significance of results, on the one hand, and to enhance the correlation analysis by clearing them from undesired side effects generated by the complexity of the interactions' structure, on the other. Henceforth, the first step that needs to be done is that of formalising a *suitable* null model for a correlation analysis of a multiplex based on a network reciprocity measure. Indeed, a null model has to preserve some features of the network that we know are relevant, but not informative for the ongoing investigation and what we suspect may affect our analysis. Figure A shows, by the means of a very simple and stylized multiplex with three nodes and two layers, to what extent the reciprocity structure within each layer can affect the observed correlation between layers (width of the arrows indicates the intensity of the flows). *Prime facie*, the red and the yellow layers seem to be very correlated when moving across nodes. Nevertheless, this is due the combined effect of the reciprocity level within each layer and the topology of the multiplex. For instance, node C is a hub in both layers and thus nodes A and B tend to exchange with C more than between each other. The entanglement of these two effects engenders an observed correlation between the two layers.

Interestingly, the degree of global symmetry of a network derives locally from the tendency of nodes to balance inflow and outflow [9]. Overall the multiplex' layers tend to be weakly balanced, yet this effect seems to be stable in time. In other words, in both the financial and in environmental networks there are net exporter/importers of

finance/matter in the OECD countries. To what extent will this topology affect our measures of correlation?

In order to clear correlation measures across layers due to the topological effects, we adopt the formalism of Exponential Random Graphs or p^* models, which allow to obtain maximally random ensembles of networks with specified constraints. Exponential random graphs were first introduced in social network analysis [6, 5, 15, 16] and then recently rephrased within a maximum-entropy approach typical of statistical physics [17, 15, 16]. Exponential Random Graphs are very useful when one needs to understand, as in our case, the expected effects of a given set of topological properties, $\vec{C}$ (such as the total weight, or the strength sequence) on the structure of networks. Recently, a method based on the maximum-likelihood principle was proposed [10] in order to fit exponential random graphs to a real-world graph $\mathbf{G}^*$ exactly [10]. This method provides null models which specify the effects of one or more constraints on the structure of the *particular* network $\mathbf{G}^*$, and hence allows to empirically detect patterns in the latter, identified as deviations from the model's predictions [10]. In this method, maximum-entropy exponential random graphs are generated by specifying an ensemble $\mathcal{G}$ of allowed graphs, and by looking for the probability $P(\mathbf{G}|\vec{\theta})$ of generating a single graph $\mathbf{G}$ in the ensemble in such a way that the Shannon entropy

$$S(\vec{\theta}) \equiv -\sum_{\mathbf{G}\in\mathcal{G}} P(\mathbf{G}|\vec{\theta}) \ln P(\mathbf{G}|\vec{\theta}) \tag{18}$$

is maximum, under the constraints that the probability is properly normalized, $\sum_{\mathbf{G}\in\mathcal{G}} P(\mathbf{G}|\vec{\theta}) = 1$, $\forall\vec{\theta}$, and that the expected value

$$\langle\vec{C}\rangle_{\vec{\theta}} \equiv \sum_{\mathbf{G}\in\mathcal{G}} \vec{C}(\mathbf{G}) P(\mathbf{G}|\vec{\theta}) \tag{19}$$

of the set $\vec{C}$ of enforced topological properties equals the particular value $\vec{C}^* \equiv \vec{C}(\mathbf{G}^*)$ observed on the real network $\mathbf{G}^*$:

$$\langle\vec{C}\rangle_{\vec{\theta}^*} = \vec{C}^*. \tag{20}$$

In the above expressions, $\vec{\theta}$ is a vector of Langrange multipliers allowing to tune the value of $\langle\vec{C}\rangle_{\vec{\theta}}$, and $\vec{\theta}^*$ is the specific value of $\vec{\theta}$ that makes $\langle\vec{C}\rangle_{\vec{\theta}}$ coincide with $\vec{C}^*$, as dictated by the maximum-likelihood principle [18]. The solution to the above constrained maximization problem is

$$P(\mathbf{G}|\vec{\theta}^*) = \frac{e^{-H(\mathbf{G}|\vec{\theta}^*)}}{Z(\vec{\theta}^*)} \tag{21}$$

where

$$H(\mathbf{G}|\vec{\theta}^*) = \vec{\theta}^* \cdot \vec{C}(\mathbf{G}) \tag{22}$$

is sometimes called the *graph Hamiltonian* and

$$Z(\vec{\theta}^*) = \sum_{\mathbf{G}\in\mathcal{G}} e^{-H(\mathbf{G}|\vec{\theta}^*)} \tag{23}$$

is the *partition function*, ensuring that the probability is properly normalized. The above formal results translate into specific quantitative expectations when a particular choice of the constraints, $\vec{C}$, is made.

Once the numerical values of the Lagrange multipliers are found, they can be used to find the ensemble average, $\langle X\rangle^*$, of any topological property X of interest:

$$\langle X\rangle^* = \sum_{\mathbf{G}\in\mathcal{G}} X(\mathbf{G})P(\mathbf{G}|\vec{\theta}^*). \quad (24)$$

The exact computation of the expected values can be very difficult. For this reason it is often necessary to rest on the linear approximation method even if, in what follows, the only approximation will be that of treating the expected value of a ratio, as the ratio of the expected values: $\langle n/d\rangle \simeq \langle n\rangle/\langle d\rangle$.

Within this framework [10, 18], the observed constraints θ are measured on the particular real network and are used to fix the model parameters to the values . This approach is similar to the ERG used in traditional social network analysis, where maximum-entropy ensembles of networks correspond to the p^*, logit or exponential random graph models [19, 20]. The maximum-likelihood parameter choice is exactly what we need in order to obtain statistically correct expectations over ensembles of randomized variants of any particular real-world network. This allows to understand which properties of a real-world network can be simply traced back to the enforced constraints. In the aforementioned techniques employed in social networks analysis the fitting of θ^* on θ is done by numerical estimations. Monte Carlo Markov chain maximum (MCMC) likelihood estimation, when available, is the preferred estimation procedure. It is noteworthy that in the present analysis θ^* is analytically resolved and it is thus possible to find the maximum-likelihood parameter values exactly without resorting to the approximate techniques [19, 20].

The next subsections will be devoted to the description of the null model appropriate for our analysis.

5.1 The Weighted Reciprocated Configuration Model (WRCM)

The financial networks are weakly reciprocated, whereas environmental networks are significantly reciprocated. As it was previously highlighted, the reciprocity structure of every layer will affect the correlation analysis of the multiplex. Therefore, it is of prominent interest for the present analysis developing a null model that incorporates both structures to filter out the combined effect of the first degree topology (import, export sequence) and the reciprocity level of every single layer. We want a null model that constrains for every node the total import, total export and the share that is mutually exchanged (reciprocated) [4]. The graph Hamiltonian thus becomes:

$$H(\mathbf{G}|\vec{\theta}) = \sum_i (\alpha_i s_i^{\rightarrow} + \beta_i s_i^{\leftarrow} + \gamma_i s_i^{\leftrightarrow}) \quad (25)$$

where, now, $\vec{\theta} \equiv \{\vec{\alpha},\ \vec{\beta},\ \vec{\gamma}\}$ and

$$s_i^{\rightarrow} \equiv \sum_{j(\neq i)} w_{ij}^{\rightarrow},\ s_i^{\leftarrow} \equiv \sum_{j(\neq i)} w_{ij}^{\leftarrow},\ s_i^{\leftrightarrow} \equiv \sum_{j(\neq i)} w_{ij}^{\leftrightarrow} \quad (26)$$

with obvious meaning of the symbols (defined above). The partition function now becomes

$$\begin{aligned} Z(\vec{\theta}) &= \prod_{i<j} \frac{(1-x_i x_j y_i y_j)}{(1-x_i y_j)(1-x_j y_i)(1-z_i z_j)} \equiv \\ &\equiv \prod_{i<j} Z_{ij}^{WRCM}(\vec{\theta}) \end{aligned} \tag{27}$$

and the likelihood is

$$\begin{aligned} \ln P(\mathbf{G}^*|\vec{\theta}) &= \sum_{i<j} [(w_{ij}^{\rightarrow})^* \ln(x_i y_j) + (w_{ij}^{\leftarrow})^* \ln(x_j y_i) + \\ &+ (w_{ij}^{\leftrightarrow})^* \ln(z_i z_j) - \ln Z_{ij}^{WRCM}(\vec{\theta})]. \end{aligned} \tag{28}$$

The solution to this optimization problem, with respect to $\vec{x}$, $\vec{y}$ and $\vec{z}$, can be found by solving the following system:

$$\begin{cases} s_i^{\rightarrow}(\mathbf{G}^*) &= \sum_{j\neq i} \langle w_{ij}^{\rightarrow} \rangle_{\vec{\theta}^*} = \langle s_i^{\rightarrow} \rangle_{\vec{\theta}^*}, \quad \forall i \\ s_i^{\leftarrow}(\mathbf{G}^*) &= \sum_{j\neq i} \langle w_{ij}^{\leftarrow} \rangle_{\vec{\theta}^*} = \langle s_i^{\leftarrow} \rangle_{\vec{\theta}^*}, \quad \forall i \\ s_i^{\leftrightarrow}(\mathbf{G}^*) &= \sum_{j\neq i} \langle w_{ij}^{\leftrightarrow} \rangle_{\vec{\theta}^*} = \langle s_i^{\leftrightarrow} \rangle_{\vec{\theta}^*}, \quad \forall i \end{cases} \tag{29}$$

where

$$\langle w_{ij}^{\rightarrow} \rangle_{\vec{\theta}^*} = \frac{x_i^* y_j^* (1 - x_j^* y_i^*)}{(1 - x_i^* y_j^*)(1 - x_i^* x_j^* y_i^* y_j^*)}, \tag{30}$$

$$\langle w_{ij}^{\leftarrow} \rangle_{\vec{\theta}^*} = \frac{x_j^* y_i^* (1 - x_i^* y_j^*)}{(1 - x_j^* y_i^*)(1 - x_i^* x_j^* y_i^* y_j^*)}, \tag{31}$$

$$\langle w_{ij}^{\leftrightarrow} \rangle_{\vec{\theta}^*} = \frac{z_i^* z_j^*}{1 - z_i^* z_j^*}. \tag{32}$$

By the definition of the WRCM model, we not only recover the result that the global reciprocity is equal to the observed one (implying $r \equiv \langle r \rangle_{WRCM}$ and $\rho_{WRCM} \equiv 0$, also valid for the WRM). Now, all the vertex-level, strength sequences are exactly reproduced, implying that the reciprocity is reproduced at a *local* level.

In what follows, we are able to filter out first order and second order topological effects, generated by a single-layer topology, from cross-layers correlations. The enhanced measures of synergic and reverse correlations will thus be: ρ-correlations (eq. 14) and μ-correlations (eq. 15). Figure B shows the correlation matrix for synergic flows and reverse flows for the entire period of investigation (temperature maps). Shades of yellow indicate a positive correlation and shades of blue negative ones, from lighter to more intense for stronger correlations. According to our analysis, the correlation between synergic environmental and financial flows is weakly positive, but persistent over time. In contrast, the correlation between reverse environmental and financial flows is less stable across time and it does not highlight a clear pattern. Environmental, both synergic and reverse, flows are highly and positively correlated within themselves. Correlation across financial synergic flows is volatile and varies from weakly positive to negative, but reverse correlation is mostly negative, despite its variation in intensity.

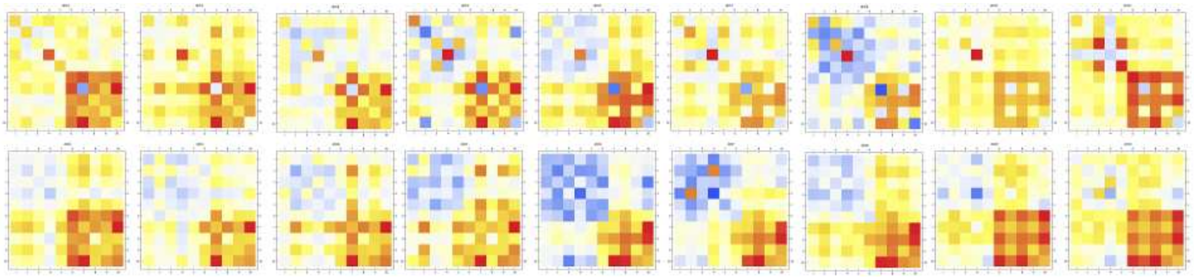

Figure B. Time evolution of the μ multiplexity correlation matrix (synergic flows), first row, and ρ reciprocity correlation matrix (reverse flows), second row, 2002-2010

6 Local reciprocity and local multiplexity

We have defined a measure of layers' reciprocity (equation 11) and multiplexity and upon that a measure of layers correlation for synergic and reverse flows of the multiplex (equations 14 and 15). This latter measure of correlation embodies a null model, the RWCM (equation 29) that preserves the topology and the reciprocity structure of each layer. Therefore, this measure enables us to observe cross correlation between layers that are not trivially produced by the overlapping of layers with different reciprocity structure. We can further extrapolate a measure of local reciprocity normalized on every column (export) for every couple of country i and j and pair of layers A and B:

$$r_{ij}^{AB} = \frac{w_{ij}^{A} w_{ji}^{B}}{\sum_j w_{ij}^{A} \sum_j w_{ji}^{B}} \tag{33}$$

and a corresponding local multiplexity:

$$m_{ij}^{AB} = \frac{w_{ij}^{A} w_{ij}^{B}}{\sum_j w_{ij}^{A} \sum_j w_{ij}^{B}} \tag{34}$$

In what follows, equation (33) enables us to compute a matrix of local, cross-layer reciprocities for a couple of layers and a couple nodes, and to use them as measures of spurious (local) correlations. This measures score 1 when all links' weights of nodes i and j, for both layers A and B, are placed between node i and j. Likewise global reciprocity across layers (equations 11 and 12) can be enhanced by incorporating the null model in the ρ and μ correlations, local reciprocity measurements can be equally improved with the use of the null model. Therefore, local ρ and μ for a couple of nodes i and j, between layer A and B are:

$$\mu_{ij}^{AB} = \frac{m_{ij}^{A} - \langle m_{ij}^{AB} \rangle}{1 - \langle m_{ij}^{AB} \rangle} \tag{35}$$

$$\rho_{ij}^{AB} = \frac{r_{ij}^{A} - \langle r_{ij}^{AB} \rangle}{1 - \langle r_{ij}^{AB} \rangle} \tag{36}$$

In section 8 dedicated to the error analysis it will be explained why negative values of ρ and μ correlations are biased (RWCM tends to overestimate negative correlations and underestimate positive correlations). In what follows, only positive correlations are truly informative. It should also be noted, that the meaning of the null model inform us when

a correlation between layers and between countries are trivially explained by single layer topology. In this case ρ or μ would score 0. Therefore a positive value of ρ or μ means that the correlations between financial and environmental layers (cross-correlations) are stronger than the single layer correlations. The null model, therefore, set a *threshold* for significant positive correlations across layers and between countries in the multiplex which is informative, as it remove the effects of the single-layer topology and reciprocity. We will thus proceed considering only positive μ and ρ correlations as significantly signed by the null model. In Fig. 3, left panel, of the main text, yellow dots signal positive correlations. Yellow lines at the far right show that some countries with a high financialisation (last columns) exchange finance with environment, prominently with countries with low financialisation (first columns). Here, correlations relate to outgoing financial flows (exports) with incoming environmental flows (imports). This correlation is expressed by yellow dots in the top-right block of the μ and ρ matrix. Conversely, countries with low finacialisation exchange mainly with countries with high finacialisation, as the yellow dots in the bottom-left block of the matrix indicates. We have replicated this exercise for all cross-layer correlation between financial and environmental layers separately and we have found the existence of a common topology among the couples of financial-environmental layers: positive correlation between each couple of outward financial and inward environmental (either synergic or reverse) flows strengthens as the level of financialization increases. In the next section, we will also investigate whether reverse correlations are more significant than synergic correlations and whether this pattern is more accentuated in reverse than in synergic correlation matrixes. All the cross-layer correlations between financial layers and environmental layers display the same distribution of yellow dots, signalling the existence of a common topological pattern in all the couple of layers. The analysis of the cross-layer correlation between the four financial layers and the five environmental layers in aggregate highlights the pivotal roles of five countries among the others. These five countries are hubs in the topology of the flows between finance and environment: UK, Germany, Bel-Lux, France and USA. This peculiar topology will be addressed in the next section.

6.1 Correlation between layers

Table C shows the average Pearson correlation index for synergic and reverse and standard deviation. Table D shows the average reciprocity and multiplexity respectively. Interestingly, reverse correlations between financial and environmental layers are generally stronger and more frequent than synergic ones. A second interesting result is the dominance of the equity market. Equities is the financial layer most correlated to environmental layers, followed by total debts securities (TD)and short term debts securities (SD). It is noteworthy that equities and debts' securities are generally considered speculative financial tools. Surprisingly, foreign direct investments (FDI), that accounts for direct, long term investments in the economy, is generally weakly correlated to the environment.

The environmental layer with the highest correlation degree to financial layers is SO_2, followed by NO_x. The production of both is generally associated with combustion and prominently with *inefficient* combustion, hinting at a specific, antiquate electricity mix, heavy industry, such, metals, metallurgy or machinery and chemical industry. However, more research is needed to clarify such links.

Table C. Average Pearson correlation of the financial flows with the environmental flows

Layer	NO_x	$PM10$	SO_2	CO_2	Water
FDI syn	0.25 ± 0.03	0.10 ± 0.03	0.28 ± 0.03	0.18 ± 0.04	0.12 ± 0.06
FDI rev	0.23 ± 0.05	0.11 ± 0.06	0.28 ± 0.05	0.14 ± 0.06	0.25 ± 0.07
Equity syn	0.32 ± 0.09	0.16 ± 0.06	0.34 ± 0.09	0.24 ± 0.12	0.27 ± 0.17
Equity rev	0.34 ± 0.15	0.15 ± 0.09	0.36 ± 0.13	0.23 ± 0.13	0.26 ± 0.10
SD syn	0.25 ± 0.05	0.08 ± 0.03	0.27 ± 0.04	0.17 ± 0.02	0.11 ± 0.05
SD rev	0.24 ± 0.05	0.12 ± 0.05	0.28 ± 0.06	0.15 ± 0.05	0.26 ± 0.09
LD syn	0.15 ± 0.03	0.05 ± 0.02	0.17 ± 0.02	0.09 ± 0.02	0.12 ± 0.04
LD rev	0.16 ± 0.05	0.05 ± 0.03	0.16 ± 0.05	0.10 ± 0.05	0.10 ± 0.04
TD syn	0.32 ± 0.09	0.13 ± 0.04	0.34 ± 0.08	0.23 ± 0.08	0.22 ± 0.15
TD rev	0.33 ± 0.12	0.16 ± 0.08	0.37 ± 0.10	0.23 ± 0.13	0.29 ± 0.10

Table D. Average reciprocity and multiplexity of the financial flows with the environmental flows, values rescaled to 10^2

Layer	NO_x	$PM10$	SO_2	CO_2	Water
FDI mul	0.45 ± 0.06	0.30 ± 0.05	0.39 ± 0.06	0.39 ± 0.07	0.32 ± 0.09
FDI rec	0.42 ± 0.07	0.33 ± 0.09	0.45 ± 0.07	0.32 ± 0.09	0.56 ± 0.13
Equity mul	0.63 ± 0.22	0.50 ± 0.17	0.62 ± 0.20	0.55 ± 0.18	0.73 ± 0.52
Equity rec	0.67 ± 0.35	0.50 ± 0.31	0.66 ± 0.29	0.56 ± 0.39	0.67 ± 0.21
SD mul	0.45 ± 0.09	0.29 ± 0.07	0.47 ± 0.07	0.38 ± 0.09	0.31 ± 0.08
SD rec	0.45 ± 0.08	0.36 ± 0.10	0.48 ± 0.08	0.34 ± 0.07	0.62 ± 0.21
LD mul	0.43 ± 0.09	0.26 ± 0.09	0.44 ± 0.06	0.32 ± 0.07	0.46 ± 0.13
LD rec	0.44 ± 0.11	0.24 ± 0.09	0.42 ± 0.09	0.34 ± 0.11	0.38 ± 0.13
TD mul	0.55 ± 0.15	0.39 ± 0.11	0.55 ± 0.13	0.47 ± 0.14	0.53 ± 0.33
TD rec	0.57 ± 0.20	0.45 ± 0.18	0.58 ± 0.16	0.47 ± 0.24	0.67 ± 0.21

6.2 Correlation between countries

In the section on local reciprocity we have proposed a methodology to investigate correlations between countries and between layers and we have shown that these measures can be implemented with the adopted null model. However, as was previously highlighted, only positive correlations are truly informative. In what follows, we can translate the correlation matrix depicted in Fig. 2 into a binary, directed matrix, by replacing positive correlations with 1 and negative or zero-correlations with 0. The binary directed matrix can be further manipulated in order to extract a binary undirected matrix, according to the relationship: $U = A * (A^T)$, where A is the binary directed matrix and U is the undirected matrix. The largest connected component of U is the backbone of the correlation network between two layers. Fig. 3 of the text shows the backbone for the financial-environmental multiplex. Hubs of the backbone are Germany (27), Bel-Lux (29), USA (33) and France (32) for synergic flows and UK (25), Germany and Bel-Lux for reverse flows. The analysis of the backbones of all the 25 pair of financial-environmental layers shows that nodes 24, 26, 30 and 31 are generally poorly connected. That is to say that, despite being highly financialized countries, Canada, Nedherlands, Australia and Israel are marginal in the correlation network of financial-environmental flows. This not

to say that they *do not exchange* either finance or environment, or both, but that are less strongly coupled to the activities of other OECD countries.

We identified the number of positive links for each country and plotted on a temporal axis to further extended the binary analysis of the topology of significant correlations to the whole multiplex, focusing on correlations between financial layers on the one hand and environmental layers on the other hand. S1 Fig. shows the time evolution of number of directional links for all OECD countries between all the couples of financial and environmental layers. The first row of S1 Fig. shows the number of incoming and outgoing (directional) links for synergic flows (μ correlations) and the second row for reverse flows (ρ correlations). Correlations always report a financial layer versus an environmental layer, thus, an outgoing link means that financial outflows are positevely correlated to the environmental footprint of imports. According to our analysis, five countries among the OECD group stick out in terms of number of links: USA (33), France (32), Bel-Lux (29), Germany (27) and UK (25). Therefore, we must conclude that there is a peculiar, common topology in all the cross-correlation networks, featured by the same five central nodes. Furthermore, it is worth noting that this topology is stable over time and robust to shocks, as it seems to have overcome the crisis of 2008 almost unaltered (with the exception of USA).

Indeed, these five countries are pivotal in the financial system and in the international trade network (ITN). A previous analysis of the correlations between financial layers and trades emphasizes their leading role [22, 23].

7 Node balancing and layers correlation

Albeit the filtering of the effects of topology and reciprocity of every single layer on the correlation between layers, node balancing (the balance between exports and imports) might still be crucial in explaining the observed patterns. The ten layers are generally unbalanced, meaning that nodes tend to be either net importers or exporters of finance or environment. Are those five central nodes in the correlation networks also hubs in the financial system and/or in the trade network? Are they net exporters or importers respectively? Predictably, these five countries are net importers of environment, with some exceptions, like USA in the water flows, Germany in PM10 and Bel-Lux in most environmental layers, albeit with a decreasing trend. It is worth noting that these five countries are also net importers of finance. Furthermore, nodes' imbalance of the financial layers seems to be more unstable compared to the environmental layers, but this is not a surprise given the volatility of the financial sector compared to the real economy (S2 Fig.) . Despite this volatility, correlations, and prominently reverse correlations, between financial layers and environmental layers are stable along time (see Table C), hinting to some broader, fundamental process underpinning the economy and linking trades to finance.

S3 and S4 Figs. show the Export (y axis) and the Imports (x axis) on a Log_{10} scale for the five financial layers and for trade in mass units. Countries are labelled with four colors indicating four degree of finanzialization, from light yellow to red. Countries' position along the principal axis shows the magnitude of their economy. OECD countries are broadly distributed along the line of balance and regardless of the size of their economy

or their financialisation, they divide themselves in net-exporter or net-importer, showing an inner, global balance, of mass and money. However, contrary to the five hubs, most of OECD countries are alternatively net importers or finance or net importers of mass and environmental load. Furthermore, countries that are net importer of environment tend to be net importer in all the environmental networks, and *vice versa*. Not the same can be said for financial layers: we do not generally witness to a position of net importers/exporters in all the layers simultaneously. It is also interesting to note that not all the least financialised countries are net importer of finance/exporter of environment likewise not all the most financialised countries are net exporter of finance/importer of environment. Furthermore, countries that are net importer/exporter of finance can be net importer/exporter of environment too, like in the case of the five hubs. The two systems indeed do not seem to level off each other.

8 Error analysis

The present analysis aims at investigating the durable and stable relationships between financial and environmental layers in OECD economies. Therefore, we trace correlations on the average values of trade flows along time. Hence, a first source of error of our analysis is the time variation of the analysis. Financial layers are much more volatile compared to environmental layers. Correlation measures involving equities (layer 2) might be significantly affected by the year 2008, which saw a dramatic drop of money in the equity market diverted to other assets. We tested correlations between 2002-2010 without the year 2008. Despite decreasing the correlation of equities to the remaining layers, overall results hold and equities along with TD are still considerably more correlated to the environment than the other financial layers.

We further assess the sensitiveness of Pearson correlations to time variation within layers by jack-knifing the time sample and evaluating the error magnitude (the variance of the jack-knife estimator). According to our error analysis, the ranking of correlations expressed in the analysis are significant. As previously highlighted, our analysis, when endowing the null model, takes into account just positive correlations. This is because the null model here described tends to overestimate negative correlations. This bias is due to the combination of two effects:1) the null model is always a full graph (though some links have small values compared to the scale of network's weights), whereas the real financial graphs are half connected; 2) the model fails to estimate links with small weights and prominently for nodes with lesser out strength (exports). The first effect is caused by a computational shortcoming: the log-likelihood maximization delivers non-negative values [10]. It should be noted that the correlation index ρ computed for every couple of nodes is negative any time a weight w_{ij} for either layer A or B is null, and thus r_{ij} will be zero. Bearing in mind that the financial layers are sparse, with an average density (connectance) of 0.5, we expect many *missing zeros* in the score of the ρ, as the expected value of $< r_{ij} >$ is always positive. By looking at the equation 14 we know that any *missing zero* will be replaced by a negative value of ρ. Furthermore, as it is clear form S5 Fig, the null model tends to overestimate links' weights. In S5 Fig. green dots overlap to red dots when real network's weights are predicted by the null model. Clearly, unmatched green dots lay mainly *under* red dots and mostly for smaller out strengths.

This is to say that the null model fails to predict small weights. Nevertheless, we should bear in mind that the meaning of a null model is not that of *predicting* a real graph given a certain, postulated information (like the import/export structure). It is rather to set a level of significance for our observations. Hence, positive correlations are *conservative* estimations of correlation: positive correlations that survive the combination of those two effects are thus more informative and trustworthy. In what follows, differently to Tables C and D, average values are computed separately on the observed and expected matrices from 2002 to 2010 and successively μ and ρ are calculated. In Table E we show results of the μ and ρ (positive) correlations (equations 14 and 15) for the five financial layers compared with the five environment layers. Missing values refers to negative correlations and are omitted. Values are averaged over time (9 years) and over the environmental layers. Results in Table C generally hold under the scrutiny of the null model. The most correlated financial layers are Equities, TD and lastly SD. Reverse flows are generally more correlated to the environment than synergic flows.

Table E. Average Pearson correlation of the financial flows with the environment ones, years 2002-2010, without 2008

Layer	NO_x	$PM10$	SO_2	CO_2	Water
FDI syn	0.26 ± 0.04	0.10 ± 0.03	0.29 ± 0.03	0.19 ± 0.03	0.12 ± 0.06
FDI rev	0.23 ± 0.06	0.11 ± 0.06	0.27 ± 0.06	0.13 ± 0.06	0.26 ± 0.06
Equity syn	0.32 ± 0.10	0.17 ± 0.06	0.34 ± 0.10	0.26 ± 0.10	0.26 ± 0.18
Equity rev	0.33 ± 0.15	0.14 ± 0.10	0.34 ± 0.13	0.21 ± 0.16	0.27 ± 0.10
SD syn	0.25 ± 0.05	0.09 ± 0.04	0.28 ± 0.04	0.18 ± 0.05	0.11 ± 0.05
SD rev	0.24 ± 0.05	0.12 ± 0.05	0.28 ± 0.06	0.15 ± 0.05	0.26 ± 0.09
LD syn	0.16 ± 0.03	0.05 ± 0.02	0.17 ± 0.03	0.10 ± 0.02	0.12 ± 0.03
LD rev	0.15 ± 0.06	0.04 ± 0.03	0.15 ± 0.05	0.09 ± 0.04	0.10 ± 0.05
TD syn	0.33 ± 0.09	0.14 ± 0.05	0.35 ± 0.09	0.25 ± 0.07	0.21 ± 0.16
TD rev	0.33 ± 0.12	0.15 ± 0.08	0.36 ± 0.11	0.21 ± 0.13	0.32 ± 0.09

Acknowledgments

We would like to thank Diego Garlaschelli, Tiziano Squartini, Francesco Picciolo and Rossana Mastandrea for their help. Without their contribution this work would have not been possible.

References

[1] Bianconi G., Statistical mechanics of multiplex networks: Entropy and overlap*Phys. Rev. E* **6**, 062806 (2013).

[2] Nicosia V., Bianconi G. & Barthelemy M. Growing Multiplex Networks. *Phys. Rev. Lett.* **5**, 058701 (2013).

[3] Barigozzi M, Fagiolo G & Garlaschelli D. Multinetwork of international trade: A commodity-specific analysis.*Phys. Rev. E* **4**, 046104 (2010).

[4] Squartini T, Picciolo F, Ruzzenenti F & Garlaschelli D. *Reciprocity of weighted networks* Sci. Rep. **3**, 2729 (2013).

[5] Holland P. & Leinhardt S. in Sociological Methodology. ed Heise D (Jossey-Bass, San Francisco), 1-45 (1975).

[6] Wasserman S. & Faust K. Social Network Analysis. Cambridge University Press (Cambridge, New York) (1994).

[7] Garlaschelli D. & Loffredo M. I. Patterns of Link Reciprocity in Directed Networks. *Phys. Rev. Lett.* **93**, 268701 (2004).

[8] Garlaschelli D. & Loffredo M. I. Multispecies grand-canonical models for networks with reciprocity. *Phys. Rev. E* **73**, 015101 (2006).

[9] Garlaschelli D., Ruzzenenti F. & Basosi R. Complex Networks and Symmetry I: a Review. *Symmetry* **2**(3) 1683-1709 (2010).

[10] Squartini T. & Garlaschelli D. Analytical maximum-likelihood method to detect patterns in real networks. *New J. Phys.* **13**, 083001 (2011).

[11] Kovanen L., Saramaki J. & Kaski K. Reciprocity of mobile phone calls. *Dynamics of Socio-Economic Systems* **2**(2), 138-151 (2011).

[12] Fagiolo G. Directed or Undirected? A New Index to Check for Directionality of Relations in Socio-Economic Networks. *Econ. Bull.* **3**(34), 1-12 (2006).

[13] Wang C., Lizardo O., Hachen D., Strathman A., Toroczkai Z., Chawla N. A dyadic reciprocity index for repeated interaction networks. *Network Science* **1**(1), 31-Âŋ48 (2013).

[14] Akoglu L., Vaz de Melo P. O. S. & Faloutsos C. Quantifying Reciprocity in Large Weighted Communication Networks. *Lec. Notes Comp. Science* **7302**, 85-96 (2012).

[15] Snijders T. A. B., Pattison P. E., Robins G. L. & Handcock M. S. New specifications for exponential random graph models. *Sociological Methodology* **36**(1), 99-153 (2006).

[16] Robins G., Pattison P., Kalish Y. & Lusher D. An introduction to exponential random graph (p*) models for social networks. *Social Networks* **29**(2), 173âĂŞ191 (2007).

[17] Park J & Newman MEJ (2004) The statistical mechanics of networks. *Phys Rev E* **70**, 066117.

[18] Garlaschelli D. & Loffredo M. I. Maximum likelihood: extracting unbiased information from complex networks. *Phys. Rev. E* **78**, 015101(R) (2008).

[19] Robins, G., Snijders, T., Wang, P., Handcock, M. & Pattison, P. Recent developments in exponential random graph ($p*$) models for social networks. *Social networks* **29**, 192-215 (2007).

[20] Wang, P., Robins, G., Pattison, P. & Lazega, E. Exponential random graph models for multilevel networks. *Social networks* **35**, 96-115 (2013).

[21] Squartini T., Garlaschelli D. & Fagiolo G. Randomizing world trade. II. A weighted network analysis. *Phys. Rev. E* **84**, 046118 (2011).

[22] Joseph A., Vodenska I., Stanley E. & Guanrong C. Netconomics: Novel Forecasting Techniques from the Combination of Big Data, Network Science and Economics: arXiv:1403.0848 [q-fin.GN]

[23] Joseph A.& Guanrong C.Composite centrality: A natural scale for complex evolving networks. Physica D **267**, 58-67 (2014).
